# LED Mini Lidar for Atmospheric Application

**DOI:** 10.3390/s19030569

**Published:** 2019-01-29

**Authors:** Tatsuo Shiina

**Affiliations:** Graduate School of Engineering, Chiba University, 1-33 Yayoi-cho, Inage-ku, Chiba-shi 263-8522, Japan; shiina@faculty.chiba-u.jp

**Keywords:** LED, lidar, atmosphere, photon counter, near range

## Abstract

The creation of a compact and easy-to-use atmospheric lidar has been the aim of researchers for a long time. Micro Pulse Lidars (MPL) and commercialized ceilometers were designed for such purposes. Laser Diodes (LD) and Diode-Pumped Solid State (DPSS) Laser technology has evolved, making lidar system more compact; however, their vulnerability to static electricity and fluctuation of electrical power prevented the growth of atmospheric lidar technology as a system suited to all kinds of users. In this study, a mini lidar with a Light Emitting Diode (LED)-based light source was designed and developed. As LED lamp modules do not need a heat sink or fan, they are resilient and can emit light for long periods with constant intensity. They also offer ease of handling for non-professionals. On the other hand, a LED lamp module has a large divergence, when compared to laser beams. A prototype LED mini lidar was thus developed, with focus on transmitting power optimization and optical design. This low-cost lidar system is not only compact, but also offers near-range measurement applications. It visualizes rapid activities of small air cells in a close range (surface atmosphere), and can verify and predict the condition of the surface atmosphere. This paper summarizes the principle, design, practical use and applications of the LED mini-lidar.

## 1. Introduction

In the field of remote sensing, near-range measurement is important in the monitoring of surrounding environments. Traditionally, contact-type sensors and transmittance meters are used to monitor such targets [1,2]. DOAS (Differential Optical Absorption Spectroscopy) is also used to monitor certain gas concentrations in air for near-range measurement, especially in horizontal directions [3,4,5]. However, measurements in several places are necessary to obtain a spatial distribution of the target. Furthermore, transmittance meters and DOAS can only offer mean pollutant concentration over a fixed path.

On the other hand, spatial distribution can be monitored by the lidar (Light Detection and Ranging) technique using pulsed lasers and by detecting backscattered light from molecules and aerosols in the atmosphere [6,7,8]. Lidar technology, herein called atmospheric lidar by the author, was developed as a calibration range finder tool in 1962 [9,10,11]. Over the past 50 years, it has seen several variations and has found practical use in atmospheric observation. The traditional lidar is mainly used to observe tropospheric atmospheres. In order to effectively transmit a high-power laser beam over a large distance of <20 km, a large laser apparatus is required. The lidar system used in such situations is thus big and consumes a large amount of power [12,13,14]. From a viewpoint of compact and easy-to-use systems, directions to downsize and make it a usable system have been explored. Micro pulse lidars (MPL), EZ lidar series (LEOSPHERE Co. Ltd.) and Laser Diode (LD) based lidars were developed for that purpose [15,16,17,18,19,20]. The former two aimed at creating an easy-to-use system, while the latter focused on it being compact. MPL now has widespread use and its network has expanded worldwide. EZ lidar has a monolithic design to make it compact and easy to use. LD lidars have several applications, the most successful of which are the ceilometer and the pseudo-Random Modulation CW lidar (RM-CW lidar). Vaisala’s Ceilometer is widely used worldwide, not only to measure cloud base, but also to observe atmosphere at low altitudes (a few kilometers) [21,22]. Random modulated continuous wave (RM-CW) lidar was the first to use a low power LD light source [23,24,25,26]. It utilized the modulated continuous wave (CW) beam of a laser diode and pseudo-random (M series) modulation, thus increasing the signal-to-noise ratio of the detected echoes. As background light cannot be negligible (because of a weak CW beam), echo intensity decreases rapidly, as compared to the usual pulse lidar. This lidar offers the advantage of near-range observation with low transmitting optical power.

Near range atmosphere/gas observations are currently in demand for a variety of situations; for example, road dust monitoring, house dust observation, aerosol-flow imaging of closed spaces (factory, gymnasium and hall), and firedamp or toxic fume detection from volcanoes, among others [27,28,29]. To install the lidar system in such near range on-site observation situations, eye safety and compactness of the lidar apparatus is essential. Furthermore, its usability and low power consumption are also important. Existing atmospheric lidars mainly utilize class 3B or 4 lasers. LD lidar has the potential to a create a low-cost system; however, maintenance of the laser diode is inconvenient, specifically in onsite settings. Non-professionals will find it hard to maintain or might not even be allowed to do so. In addition, the laser apparatus has restrictions on use at airports, chemical factories, nuclear plants, and commercial facilities.

The author focuses on Light Emitting Diode (LED) lamps as a lidar light source for atmospheric observation. LED light sources are cheap, resilient against static electricity, and offer a wide wavelength selection. A variety of LEDs with brightness of more than 10,000 mcd or output power of more than 1 W are sold in the market [30,31,32,33]. An LED lamp is easier to maintain than an LD; anyone can replace/fix it without care for electric surges or major optical alignment. LED lights can be used anywhere, as they do not have a central focal point. An LED module is a free import/export item, and there are no restrictions on the use of LED light; laser beams, however, are limited, especially in enclosed spaces. As the heat emitted from LED lamps is less and their usage is simple, an LED lighting module is small. High-repetition oscillation is easy to achieve as well. A large amount of power consumption in a lidar system is due to the laser driver, and an LED light source can help reduce it. However, LED light is difficult to collimate, as a result of which its illuminating power decreases rapidly with respect to the propagating distance. To use an LED lamp as a lidar light source, its transmitting power, light collimation, and pulse characteristics should be taken into consideration and studied.

In this study, an LED mini lidar was designed and developed for feasibility study. Its compact and light-weight apparatus has the potential to monitor the quick activity of the surface atmosphere or a certain gas in the near range. Initially, a theoretical calculation was made to estimate the minimum output pulse power for the LED light module in order to get enough signal-to-noise ratio of the atmospheric lidar echo in the near range. Pulse characteristics of the LED light source was examined and adequate conditions in the viewpoint of lidar light source was obtained. Thus, a specialized LED self-pulse driver was developed. The practical lidar system was set up with an LED-pulse module. High-speed and high-resolution photon counter was also developed to follow the high pulse repetition frequency of the LED light. Several results of the atmospheric activity were examined. The application and adaptation of the LED mini-lidar was discussed as well.

## 2. Numerical Analysis for LED Mini-Lidar Design

The LED mini-lidar concept studied in this work is for onsite use in closed and local spaces. In order to ensure that it is portable, the system needs to be compact and lightweight. Resilience against electrical noises, static electricity, and rough treatment is an important feature for onsite operation. The laser diode is tricky to control, especially when used with high pulse power outdoors, and amateurs find it hard to make optical alignments or change/fix the device. On the other hand, an LED light source is sturdy, and can easily be handled by non-professionals. An LED light source has a large divergence, and the beam aperture is large enough to collimate properly. This wide divergence enhances the overlap function at very close range, thus minimizing incident intensity for the human eye.

A numerical analysis was first performed to design and estimate the performance of the LED mini-lidar. Pulse power and observation range were fixed at >100 mW and 100–300 m, respectively. The average power of the LED beam was less than 1 mW, equivalent to a Class 1 laser. These values were defined taking into consideration eye-safety and near-range observation in closed spaces. Here, a coaxial-type optics was selected for close-range monitoring.

**Figure 1:**
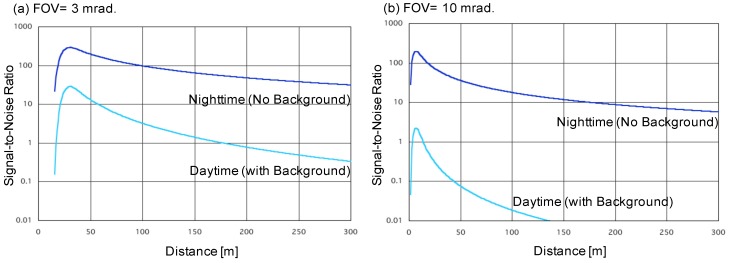
Signal-to-noise ratio of LED mini-lidar simulation.

If LED pulse with peak power *P*_0_ and pulse width *τ* is transmitted, the received power *P*(*R*) from a distance *R* is given by lidar Equation (1) [34]:

(1) $$\begin{array}{l} P\left(R\right)=P_{0}\cdot K\cdot Y\left(R\right)\cdot A_{r}\cdot \frac{c\tau }{2}\cdot \beta \left(R\right)\cdot \frac{1}{R^{2}}\cdot T{\left(R\right)}^{2}\\ T\left(R\right)=\exp\left(-2\int_{0}^{R}\alpha \left(x\right)dx\right) \end{array}$$

where *c* is the speed of light, *K* is the efficiency of receiving optics, *Y*(*R*) is the overlap function, *Ar* is the effective receiver area, *β*(*R*) is the backscattering coefficient, *T*(*R*) is the transmittance and *α*(*x*) is the atmospheric extinction coefficient. The signal-to-noise ratio (SNR) equation for the echo intensity is given as:

(2) $$SNR=\frac{\sqrt{M}\sqrt{\eta_{Q}\Delta t/h\nu }P\left(R\right)}{\sqrt{\mu }\sqrt{P\left(R\right)+Pb+Pd}}$$

where *M* is the accumulation number, *Q* is the quantum efficiency of a detector, Δ*t* is the sampling time, *h* is the Planck’s constant, *ν* is the pulse repetition frequency, *μ* is the noise factor of a detector (Photomultiplier: PMT = 1, Avalanche Photo Diode: APD = 2–3), *Pb* is the background light and *Pd* is the equivalent noise of a detector or amplifier. By using these equations, the echo intensity of the LED lidar was estimated and its specification fixed. The numerical parameters of the lidar components and atmospheric parameters are summarized in Table 1. The numerical analyses were conducted using two field of view (FOV) measurements: 3 mrad as the usual (laser based) lidar setup, and 10 mrad for a wide FOV. The estimated results are shown in Figure 1. Daytime background light power *Pb* was calculated by *Pb* = *S*Δ*λA_r_Ω*. Here, *Sr* is the luminance of background light. Δ*λ* is the optical bandwidth. *A_r_* and *Ω* are the aperture and the solid angle of the lidar optical receiver. The 3 mrad FOV case is shown in Figure 1a. The lidar echo has enough signal-to-noise ratio in the near range of up to 300 m in daytime and nighttime observations. The full overlap distance was 35 m. On the other hand, for the wide FOV (10 mrad) case, as shown in Figure 1b, the observation range can be up to 300 m at night, but reduces to <150 m in the day. The full overlap distance was shortened to less than 10 m because of a wider beam divergence and receiver’s FOV.

## 3. LED Light Source

In general, an LED has the same quick pulse response as a laser diode (LD); however, the former has a broader wavelength variety. LEDs usually offer attractive specifications of brightness (illuminance) and wavelength. Its responsivity, especially quick response (sub-nano seconds), which is utilized in lidar research, are not required and announced officially. This responsiveness depends on the LED’s materials and structures. The author examined the pulse oscillation properties of lamp-type LEDs and high-power LEDs with various wavelengths. A lamp-type LED consists of a diode chip on electric poles and an epoxy lens. Lamp-type LEDs with lens diameter of 3 mmφ or 5 mmφ were chosen for the study. A high-power LED consists of an array of diode chips and an epoxy lens.

As an ordinary lidar light source (laser-based atmospheric lidar), its duty ratio is not high (1/10^4^–1/10^5^)—its pulse width is 10–50 ns and repetition frequency of pulse oscillation is a few kHz in the case of Diode-Pumped Solid State (DPSS) laser and LD. The LED light source, however, needs to have a higher repetition frequency because of its low pulse power, and output power needs to be more than 100 mW. In addition, the duty ratio should be restricted to less than 0.01 to protect the breakdown of the LED chip due to heat. Nevertheless, repetition frequency can be up to 1 MHz, ensuring its maximum detection range >150 m at that frequency. For surface atmosphere observation, its activity depends on altitude, that is, at a lower altitude, air moves faster. Therefore, the LED lidar should have a higher spatial resolution of about 1 m, which comes from an observation height on the ground in a horizontal direction. To obtain a high range resolution of a few meters, it is necessary to shorten the pulse width to less than a few tens of nanoseconds—when the pulse width is 10–50 ns, the range resolution is 1.5–7.5 m.

Table 2 shows the correlation between the center wavelength and the minimum pulse width of lamp type LEDs (3–5 mmφ OptoSupply High luminous LEDs, OptoSupply, Hong Kong, China) and high-power LEDs (LUXEON K2 Emitter series, LUMILEDS, San Jose, CA, USA). Pulse widths are the shortest values in its pulse power. All of these LEDs offer quick response capabilities and can thus function as a lidar light source. The pulse width of the short-wavelength LED is shorter than that of the long-wavelength one. This reflects the materials and semiconductor layer structures used in the LED chips. The pulse shape of a lamp-type LED with wavelength of 392 nm could result in a 10-ns pulse width. A high-power LED has a longer pulse width than a lamp-type one, which is a summation of a series of diode chips in a module. The high-power LEDs (LUXEON K2 Emitter series), however, have a higher average power output. Their average power is more than 350 mW (@1A), and pulse width is 30–50 ns. The pseudo-random modulation lidar technique works when the average light source power is high, as it needs average power of more than 30 mW (ave.) [34]. Therefore, a high-power LED can be useful as a light source for the pseudo-random modulation lidar.

**Figure 2:**
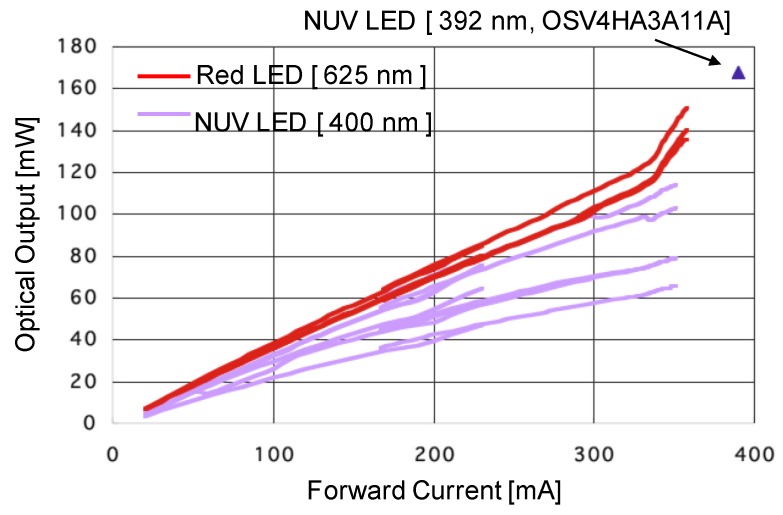
Peak powers of LED pulsed oscillation.

Figure 2 shows the relationship between the forward pulse drive current and the peak power of lamp-type LEDs; red-LED (625 nm: OptoSupply OSR5MA5111A-VW, OptoSupply, Hong Kong, China) and NUV-LED (392 nm: OptoSupply OSV4HA3A11A, 400 nm: OptoSupply OSSV5111A, OptoSupply, Hong Kong, China). The input pulse signals are 10 ns pulse wide with repetition of 100 kHz for NUV-LEDs, and 60 ns pulse wide with repetition of 100 kHz for the red LED. Each LED has different peak powers, even if they have the same model number. However, the maximum peak power was 100 mW. The output powers of the LEDs are designed taking into consideration the sensitivity of the human eye.

These results classify two types of LEDs as lidar light sources: The lamp-type LED that has a short pulse width and low average power, and the high-power LED with a high average power (even though its pulse width is longer than that of the lamp-type LED).

**Figure 3:**
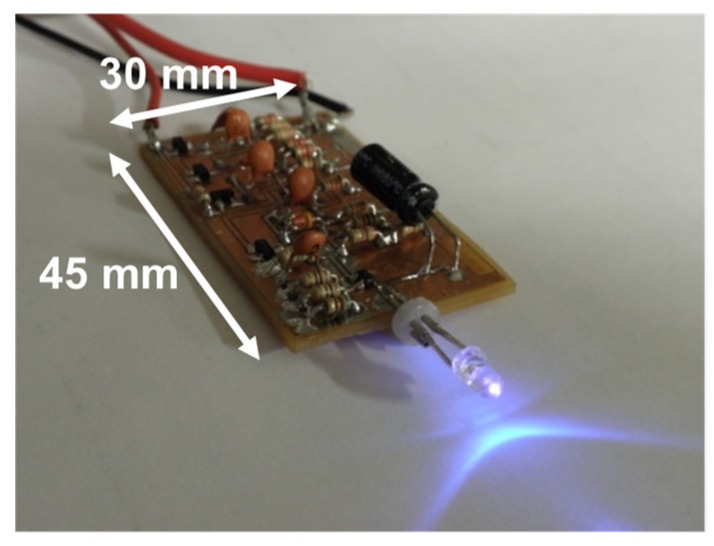
High-repetition LED pulse driver.

The oscillation signal that drives an LED lamp is produced by a combination circuit, which consists of an astable multivibrator circuit, a monostable multivibrator circuit, and an LED current driver circuit. The astable multivibrator circuit controls pulse repetition frequency, and the monostable multivibrator circuit decides pulse width. It is defined as the full-width half-maximum (FWHM) of the optical pulse shape of LED light. The developed driver circuit has adequate characteristics to enable LED light sources in lidar application. The pulse current is enough to drive a 100 mW pulse peak power. Furthermore, its pulse width is 10 ns (which is equal to a range resolution of 1.5 m), and pulse repetition frequency is 112 kHz. An illustration of the high-repetition LED pulse driver is shown in Figure 3. The dimensions of the circuit are 30 × 45 mm (L × W). In the case of the lamp-type LED with 392 nm wavelength, pulse power was ≥100 mW, and the forward current of the LED chip was about 380 mA (pulse).

## 4. Setup of LED Mini-Lidar

**Figure 4:**
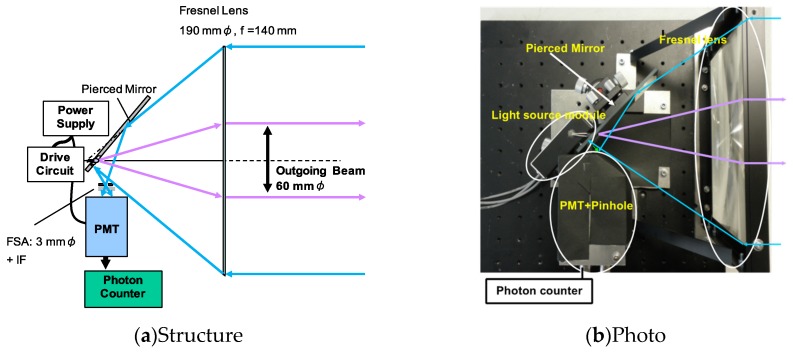
Optical layout of the LED mini-lidar (Type 1).

By taking into consideration the theoretical estimations and pulsed oscillation characteristics of the LED lamp, we developed a prototype of the LED mini-lidar. A lamp-type LED with 3 mmφ (392 nm, OSV4HA3A11A) was selected to ensure compactness of the lidar system. The focal length of the lens was 150 mm, and the LED beam was collimated at the divergence of 9.5 mrad with a beam size of 60 mmφ. The numerical analysis found that in order to obtain atmospheric echo from a close range of more than 100 m for indoor and nighttime observations, the receiver’s aperture had to be 200 mmφ. Coaxial optics was installed into the lidar optics. A single Fresnel lens was applied to transmit and receive optics—the center of the lens was used to transmit and the surrounding area was used to receive. Optical alignment was easy with regards to transmission and detection optics by a single lens. The Fresnel lens is lightweight, has a shorter focal length, and does not need severe alignment. To separate the transmitting beam and the receiving echo, a pierced mirror was installed. The schematic diagram and illustration of the LED mini-lidar are shown in Figure 4. Because the lamp-type LED has an epoxy lens, it is placed inside the focal length of the Fresnel lens. This is the result of a combination of the epoxy lens and the Fresnel lens. Installing a pierced mirror to separate the echo from the transmitting beam at the optical axis enabled the lidar to have a coaxial optic on using the single Fresnel lens [35].

Table 3 shows the specifications of the LED mini-lidar (Type 1). The effective diameter of the Fresnel lens is 190 mmφ. The pulse power of the LED light source is 120 mW. The detector was a photomultiplier tube (PMT Hamamatsu R6350P, Hamamatsu Photonics, Hamamatsu, Japan). The PMT was used in analog mode for hard target detection and in photon-counting mode for the atmospheric observation. An interference filter with bandwidth of 11 nm was placed in front of the PMT. A pinhole of 3 mmφ was installed to limit the receiver’s FOV to 10.7 mrad. The beam divergence was about 10 mrad and the overlap between the beam divergence and receiver’s FOV was 5 m ahead. The total size of the LED mini-lidar is 230 × 230 × 210 mm (L × W × H), which includes optics, the PMT unit, and power supply. The LED lidar is easy to carry because it is lightweight <2 kg. Its size is also quite compact in comparison with traditional lidar systems.

## 5. Observations

**Figure 5:**
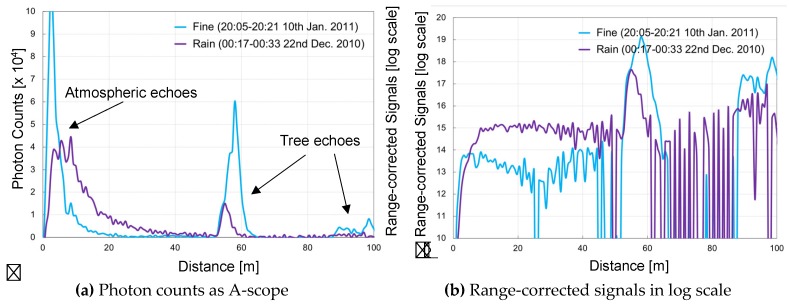
Atmosphere observations results with multi-channel scaler (SRS SR430).

Figure 5 shows the atmospheric measurements taken by the LED mini-lidar at night. The measurements were conducted in a horizontal direction. Here, the PMT was propelled in the photon-counting mode with a multi-channel scalar (SRS SR430). The accumulation was about a million times. SR430 had the maximum repetition frequency of 1.6 kHz and its time resolution was 5 ns (0.75 m) as its BIN width. It was unable to follow the higher pulse repetition frequency of the LED mini-lidar, and the scaler chose a trigger in its timing rate, in place of LED repetition frequency. As a result, the summation time to obtain a certain signal-to-noise ratio of the atmospheric echoes was 16 min. The measurements were conducted on a fine day and a rainy day. In Figure 5a, the peaks near 60 m and 100 m are tree echoes. The atmospheric echoes were captured in 0–80 m and they gradually weaken due to their inverse proportionality to the square of the propagated distance. The numbers of scattered photons on the rainy day were larger than those on the sunny day because of the backscattered light from raindrops. The nearest peak of 0–5 m on the rainy day was smaller than that on the sunny day, which is a result of the strong scattering echoes of rain droplets at the closest distance. In Figure 5b, the range-corrected echo counts are shown in log-scale. Here, the change in atmospheric conditions are clearly visualized. Both measurements were conducted on surface atmosphere in a horizontal direction. When aerosol activity is high at a low altitude, the lidar echo reflects on the surface structure and conditions of the surface atmosphere. The echo activity of the fine day reflected the condition of the surface atmosphere. On the contrary, the echo curve of the rainy day became monotonous, which was a result of constant rainfall-subsided aerosols. These results matched with the estimated results of observation range, signal-to-noise ratio, and summation time. These experimental results verified the applicability of LED lidar in near-range atmosphere measurements.

**Figure 6:**
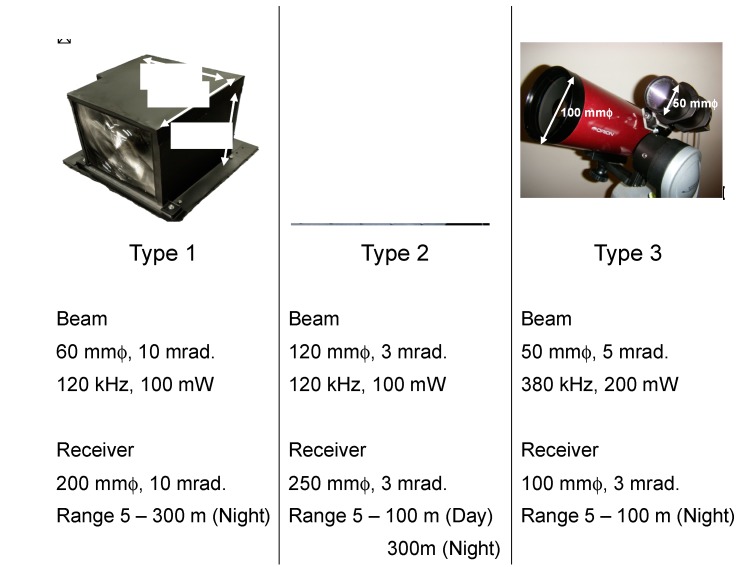
LED lidars’ variation.

After the first approach (Type 1), improvements were made to some specifications of the LED mini-lidar; they are summarized in Figure 6. Type 2 has an enlarged Fresnel lens. Its transmitting beam diameter is 120 mm and the receiver’s aperture is 250 mm. As a result, the beam divergence and receiver’s field of view are restricted to 3 mrad. The observation range is 300 m at night and 100 m in the day. They matched with the theoretical calculations. Other parameters are the same as Type 1. On the contrary, Type 3 has biaxial optics for the transmitter and the receiver. The beam diameter and its divergence are 50 mm and 5 mrad, respectively. The receiver’s aperture is 100 mm and its field of view is 3 mrad, that is, the optical efficiency between the transmitting and receiving energies is <30%, while rigorous optical alignment is not required. Type 3 demonstrated appraisable results. The transmitting power is 200 mW and its pulse repetition frequency is 380 kHz. The observation range was up to 100 m at night.

To utilize the high-repetition frequency of LED pulse oscillation, an FPGA-based high-speed photon counting board was designed and developed with the help of Trimatiz Co. Ltd. The board has a 4-channel input, making it convenient for multi-channel observation, such as orthogonal polarization measurement, Raman scattering observation for certain gases, and multi-wavelength detections. It can be synchronized with pulse beam oscillation by the trigger-in port. On the contrary, the board can also generate a trigger signal to fire the pulse beam.

**Figure 7:**
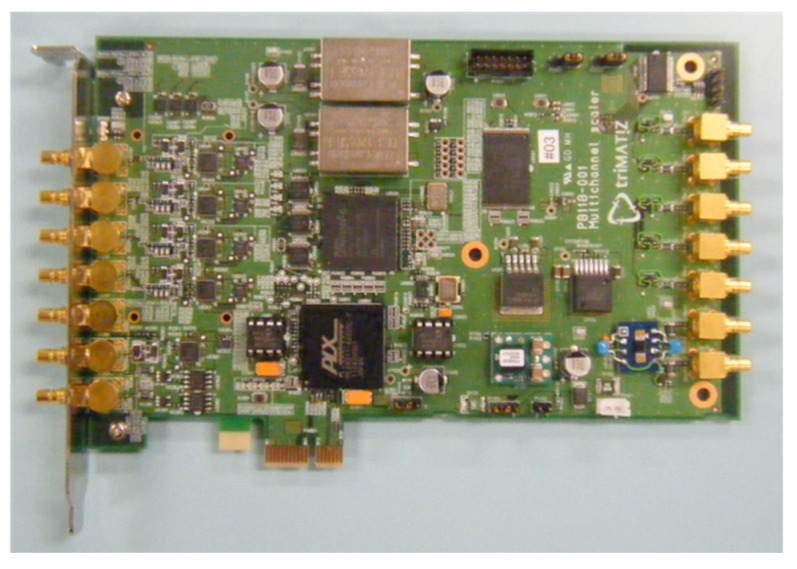
Photon counting device (Trimatiz Co. Ltd, “Photon Tracker”).

The system clock is of 500 MHz. Power consumption is 7 W, that is, <2 W for each channel. The board’s features and specifications are shown in Figure 7 and Table 4. The bin width and number, repetition frequency, signal threshold to count and trigger levels can be adjusted. The highest resolution defined by the BIN width is 5 ns, which is equal to a range resolution of 0.75 m. The minimum summation time, up to data transfer to a PC, is 0.2 s. The short summation time can capture atmospheric and gas activities.

**Figure 8:**
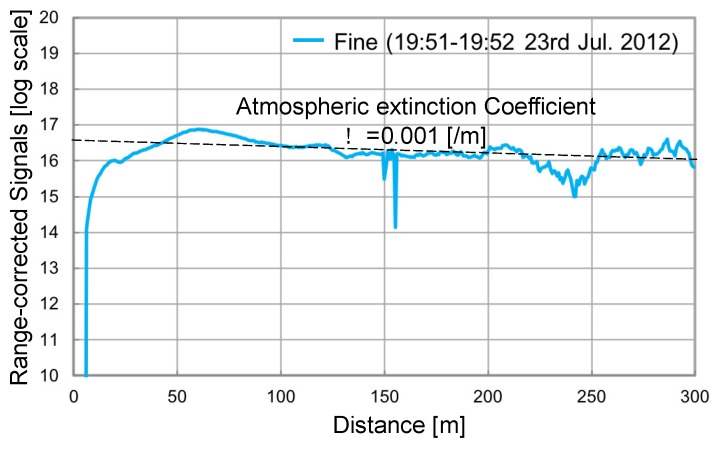
Longer range observation up to 300 m (Type 2).

The Type 1 and Type 2 LED lidars enabled longer range observation. The high-speed photon counting board was used for atmospheric activity observation. The summation time was shortened with Type 2 because of the narrower beam divergence and receiver’s FOV, both of 3 mrad. It took more than three minutes with Type 1 to get the atmospheric echoes up to 300 m, but Type 2 shortened it to <1.5 min. Figure 8 gives details of the longer-range observation (up to 300 m) obtained by Type 2. The vertical axis indicates the range-corrected signals in log scale. The summation time was 100 s. The weather condition was fine. The observation was conducted in a horizontal direction. The activity of the surface atmosphere was observed with a short summation time. The average atmospheric extinction coefficient was estimated as *α* = 0.001 [/*m*].

**Figure 9:**
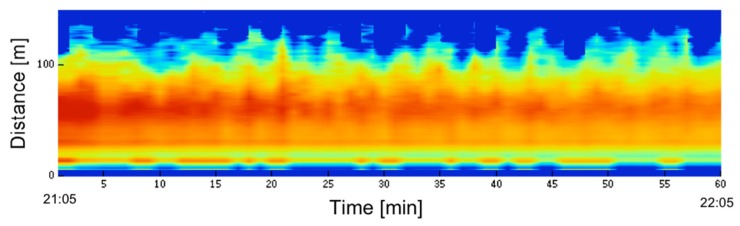
Atmospheric observation. 21:05–22:05 21th Sep. 2012. Accum.10 s. (Type 3).

The Type 3 LED mini lidar was also used for activity monitoring of the surface atmosphere, as shown in Figure 9. The observation was conducted in a horizontal direction. To obtain images, summation time was set at 10 s. Continuous observation of one hour was visualized. The horizontal axis denotes time and the vertical axis shows the distance. Photon counts were represented by fake colors, by multiplying the square of the distance and taking its log-scale (range-corrected signal in log-scale). The weather conditions were clear, and atmospheric activity was low; however, small changes in the image can still be seen. Atmospheric echoes were monitored up to 100 m. When the summation time was increased to 30 s, the observation range was increased to 300 m for nighttime observation. Even in the day, the threshold was adjusted—the observation range was reduced to 100 m under the condition that the lidar be installed in a shade place.

**Figure 10:**
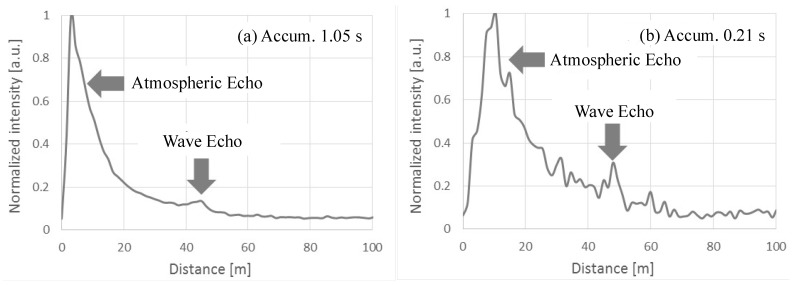
Short time observation of atmospheric activity. (Type 3).

The observation results of sea surface atmosphere are shown in Figure 10. They were created with integration time of 0.25 s. The atmospheric echo was monitored from 10 m to 80 m, while the sea wave echo was obtained at 40 m ahead. On continuing the observation, activities of the sea wave motions and surface atmosphere were captured. Figure 10 indicates changes of the alternative lidar echoes against the summation time. Here, high-resolution photon counter was utilized [36]. Its time resolution was 1 ns, which is equivalent to a distance of 0.15 m. The summation times were: (a) 1 s and (b) 0.2 s, respectively. The observations were conducted at a sea coast with a shallow elevation angle and were the results of a synchronized observation of surface atmosphere and sea wave activities. Smooth echoes are obtained in Figure 10a. The atmospheric echoes reduced in number with respect to their inverse proportionality to the square of the distance; the sea wave echo was smooth as well. It represents the wavelength of the wave. On the contrary, the activity of the atmosphere and sea wave were captured at the instantaneous value of 0.2 s (Figure 10b). The undulating echo in the atmosphere was not noise, but disturbances. The observation height was 1–2 m; the atmosphere activates with the same cell size. Its activity was visualized as well. Sea wave echo represented its instantaneous condition, that is, its activities were visualized through continuous observation in a short period of 0.2 s.

## 6. Conclusions

The LED mini-lidar was designed and developed for near-range measurement. Theoretical calculations found that pulse power >100 mW can measure atmospheric echo in the observation range of up to 300 m with enough repetition frequency of >100 kHz. As an initial approach, it was found that a 3-mm LED lamp with 392 nm, 400 nm and 625 nm wavelengths produced enough pulse power (over 100 mW), and a short-wavelength LED can reduce its pulse width to around 10 ns. By placing a pierced mirror at the optical axis, the main lens (Fresnel lens) could be utilized for both the transmitter (center section) and the receiver (outer section). As a result, the LED mini-lidar had coaxial optics to accomplish near-range measurements. This system is compact—about 20-centimeter cubic box and lightweight (<2 kg). The result of the experiments verified that the LED lidar could be utilized for short-range observation from 0 m to 100 m. The results were in agreement with the authors’ estimations. The observation range reaches 100 m in the day and 300 m at night, at an accumulation time of a few tens of seconds. The biaxial-type LED lidar was designed to demonstrate a wider beam divergence and narrower receiver’s FOV. Through the theoretical approach and practical experiments, the feasibility of the LED mini-lidar was validated.

Other applications of the LED lidar include fluorescence measurement with an NUV-LED, discrimination of a certain gas target by the Raman shifted echo detection or the absorption of a certain wavelength, and multipoint measurements by scanning LED lidars in a closed space, among others [37,38].

## Figures and Tables

**Table 1:** Specification of LED mini-lidar on atmospheric echo estimation.

**Lidar Optical Parameters**	
LED pulse power	$P_{0}=100\;\mathrm{mW}\;@\;1\;\mathrm{nJ}/10\;\mathrm{ns}$
Optical efficiency	K=0.5
Overlap distance	5 m (depends on alignment)
Aperture size	$D_{i}=0.2\;m$
Aperture area	$A_{r}={\left(D_{i}\;/\;2.0\right)}^{2}\times \;\pi {\;m}^{2}$
Number of summations	M=100,000
Sampling time	$D_{t}=10^{-9}\;s$
Planck coefficient	$h=6.63\;\times \;10^{-34}\;J\cdot s$
LED center wavelength	$\lambda_{0}=394.0\;\times \;10^{-9}\;m$
LED spectrum width	$\Delta \lambda =10.0\;\times \;10^{-9}\;m$
Optical frequency	$m_{0}=3.0\;\times \;10^{8}\;/\;\lambda$
Quantum efficiency	η=0.2
Pulse length	$2l_{0}=1.5\;m$
Noise factor	μ=1
**Atmospheric Parameters**	
Background cross section	$\beta =3.40\;\times \;10^{-6}\;/m$
Extinction coefficient	α=50.0 × β
Background light	$P_{d}=6.4\;\times \;10^{-10}\;W$
Electrical noise	$P_{d}=0\;\;W$

**Table 2:** Minimum pulse widths of various-color LEDs.

LED Type	Lamp-Type LED									High-Power LED	
Wavelength (nm)	392	400	470	505	525	590	625	850	455	505	530
Pulse width (ns)	10.0	8.15	11.15	7.4	10.2	47.6	48.1	29.28	33.2	36.4	44.5

**Table 3:** Specifications of the LED mini-lidar (Type 1).

**Transmitter**	
Light source	Lamp type LED
Model number	OptoSupply OSV4HA3A11A
Wavelength	392.5 nm (390–395 nm)
Spectrum width	11 nm
Pulse width	10.2 ns
Pulse power	120 mW
Beam size	60 mmφ
Beam divergence	9.5 mrad
Repetition rate	112 kHz
**Receiver**	
Detector	Photomultiplier tube
Model number	HAMAMATSU R6350P
Aperture diameter	22 mmφ
Pinhole	3 mm
FOV	10.7 mrad
Interference filter	OPTO-LINE FF01-395/11-25
Bandwidth	11 nm (Center 395 nm)

**Table 4:** Specification of FPGA photon counting board.

FPGA device	Spartan 6
System clock	550 MHz
BIN width	5 ns–10.486 ms [5 ns × 2n (n = 0–21)]
Number of BINs	32767
Maximum counts	32767
Input channel	1–4 (expands to 8–12)
Repetition frequency	>300 kHz
Interface	PCI Express
